# Systematic identification of functionally relevant risk alleles to stratify aggressive versus indolent prostate cancer

**DOI:** 10.18632/oncotarget.24400

**Published:** 2018-02-05

**Authors:** Salpie Nowinski, Aida Santaolalla, Ben O’Leary, Massimo Loda, Ayesha Mirchandani, Mark Emberton, Mieke Van Hemelrijck, Anita Grigoriadis

**Affiliations:** ^1^ Cancer Bioinformatics, Innovation Hub, Guy's Cancer Centre, King's College London, London, UK; ^2^ Translational Oncology & Urology Research, King's College London, London, UK; ^3^ Breast Cancer NOW Centre, The Institute of Cancer Research, The Royal Marsden Hospital, London, UK; ^4^ Department of Oncologic Pathology, Dana-Farber Cancer Institute, Harvard Medical School, Boston, MA, USA; ^5^ Division of Surgery and Interventional Science, University College London, London, UK

**Keywords:** prostate cancer, active surveillance, GWAS, functional risk alleles, somatic copy number aberrations

## Abstract

Novel approaches for classification, including molecular features, are needed to direct therapy for men with low-grade prostate cancer (PCa), especially men on active surveillance. Risk alleles identified from genome-wide association studies (GWAS) could improve prognostication. Those risk alleles that coincided with genes and somatic copy number aberrations associated with progression of PCa were selected as the most relevant for prognostication.

In a systematic literature review, a total of 698 studies were collated. Fifty-three unique SNPs residing in 29 genomic regions, including 8q24, 10q11 and 19q13, were associated with PCa progression. Functional studies implicated 21 of these single nucleotide polymorphisms (SNPs) as modulating the expression of genes in the androgen receptor pathway and several other oncogenes. In particular, 8q24, encompassing *MYC*, harbours a high density of SNPs conferring unfavourable pathological characteristics in low-grade PCa, while a copy number gain of *MYC* in low-grade PCa was associated with prostate-specific antigen recurrence after radical prostatectomy.

By combining GWAS data with gene expression and structural rearrangements, risk alleles were identified that could provide a new basis for developing a prognostication tool to guide therapy for men with early prostate cancer.

## INTRODUCTION

Prostate cancer (PCa) is the most common cancer in men [1], with around 160,000 new cases each year in the USA alone [2]. Severity is conventionally assessed using Gleason score, prostate specific antigen (PSA) levels and tumour volume [3]. Approximately 80% of men in the US with PCa are diagnosed with stage I localised disease many of whom also have a low to intermediate risk of progression [4]. Men who present with low-risk PCa as defined by a Gleason score of 6 (3+3) and whose life expectancy is thought to be at least 10 years, are usually managed through active surveillance (AS) [5, 6]. AS aims to reduce over-treatment through monitoring the disease, with the requirement for radical intervention assessed with regular PSA-tests and biopsies rather than intervening at diagnosis [7]. The patient on AS can then be redirected to curative treatment in the event of short PSA doubling time [8], deteriorating histopathological factors on repeat biopsies or other factors, such as anxiety from living with an untreated cancer. Approximately 33% of men with low-grade tumours managed on AS upgrade to a higher Gleason score within 5 years [9]. At present, there are no means of identifying which patient will progress; therefore, there is an urgent need to improve risk stratification. Molecular markers may help to discriminate indolent and aggressive clinical phenotypes, and inform patient stratification for men with low-risk PCa on AS.

### Gene expression-based companion diagnostics for low-grade PCas

Gene expression profiling of PCa may help to stratify patients into high and low risk for disease progression [10]. Three commercially available tests, namely Prolaris® by Myriad Genetics (Salt Lake City, UT, USA) [11], Oncotype DX®, by Genomic Health (Redwood City, CA, USA) [12] and Decipher Biopsy™ by GenomeDx Biosciences (San Diego, CA, USA) [13, 14] are primarily focused on measuring the expression levels of genes involved in proliferation [14]. As in many cancers, expression of such genes can be used as a proxy indication of aggressive tumour cells [15].

### Germline single nucleotide polymorphisms as risk factors

A further potential, non-invasive, avenue of identifying progression in men with low-grade PCa, could be the investigation of susceptibility single nucleotide polymorphisms (SNPs), obtained from a simple blood test. Several genome-wide association studies (GWAS) have reported positive family histories [16] with an associated 2-to 4-fold increased risk of developing PCa [17]. The Stockholm 3 Model (STHLM3) can predict if a patient has a Gleason score ≥7 [18] and has been shown to have an increased sensitivity of 20% over current clinical markers in a Swedish population [19].

### The PCa genome

Even in localised, non-indolent PCa, a striking inter-tumoural heterogeneity exists at the molecular level [20], including tumours with complex structural rearrangements such chromothripsis and chromoplexy [21]. The PCa genome is thought to evolve in abrupt, periodic bursts, resulting in complex, large-scale reshuffling of the genome, known as punctuated evolution. Most of these complex rearrangements are thought to occur as early events [21]. It follows that genomic alterations present in localised low-grade PCa may provide indicators of future progression, perhaps increasing the functional effects of germline SNPs which could be regarded as *“hard-coded”* markers for the stratification of men on AS with low-risk PCas [21, 22].

These “hard-coded” markers could help circumvent the principal challenge of molecularly characterising PCa – multiple, heterogeneous tumour foci. Biopsies have a 23–46% likelihood of sampling errors with an increased chance of missing a higher grade or higher-stage tumour [23]. If a higher-grade carcinoma is detected through repeat biopsies in a man on AS, it is unknown whether this is the biological progression of the original low-grade PCa or a pre-existing clone which has been missed in the initial biopsy.

Here, we systematically collected diverse molecular data associated with aggressiveness and progression of PCa to highlight potential risk alleles. Due to the paucity of published low-grade GWAS studies, high-grade PCa GWAS studies were also included. We initially assessed germline SNPs with known associations for disease progression and aggressiveness in PCa, to identify potential genomic loci. Since somatic aberrations can influence germline changes, and vice versa [24], we then integrated available data on the functional roles of these SNPs, as well as copy number and gene expression studies to assess the potential relevance of these loci for the aggressive progression of low-grade PCa.

## RESULTS

A total of 22 PCa GWAS, and GWAS validation studies across different populations were used to identify SNPs potentially associated with aggressiveness, progression, biochemical recurrence, and metastasis. Five of the 22 studies focused on both identifying novel risk variants and validating previous findings (>1,500 SNPs) [16, 25–28]. Two studies were GWAS meta-analyses [29, 30], and 17 studies primarily sought to validate previously identified SNPs (<100 SNPs) [31–45].

Fifty-three unique SNPs were determined from these studies, residing in 29 unique loci across the genome (Supplementary Table 1). Nine of these SNPs were significantly associated with aggressive disease or unfavorable characteristics in several population studies, *e.g.* rs10993994 [16, 44, 36, 40, 41] was significantly associated with aggressiveness in Asian Indian, Ashkenazi, Taiwanese, European, African American, Australian, Canadian and US populations (Supplementary Table 1). Three SNPs were reported in three or more studies (Supplementary Table 1), rs10993994, rs1447295, and rs2735839. All three SNPs were at cytobands with other SNPs also found to be associated with aggressive PCa: rs10993994 at 10q11.23 harbouring rs7920517, rs1447295 at 8q24.21 located along with seven other SNPs, and rs2735839 located along with three other SNPs (Supplementary Table 1) No SNPs in high linkage disequilibrium with rs2735839 were found to be associated with PCa [46].

A total of three low-grade and low-volume-tumour studies [5, 37, 43] were initially selected from the search criteria. Only two of these were finally selected through the inclusion criteria [37, 43] (Supplementary Figure 1). The first study was carried out on radical prostatectomies of AS patients where unfavourable pathological characteristics were measured [37] and corroborated a risk allele rs1447295, which was also found to be associated with aggressive PCa by three other studies [45, 44, 41]. The second study involved a comparison between men who upgraded from Gleason 6 with those who remained stable [43]. Together, these studies, reported 5 SNPs, where 2 were found in the same loci as SNPs identified in more aggressive studies, rs1447295 on 8q24 and rs11228565 on 11q13.3. The third low-grade study by Goh *et al*, [5] conducted a prospective study of 412 men on AS where 56 patients histologically upgraded upon repeat biopsies. All 39 SNPs, including rs1447295, 10993994, and rs2735839, analysed in this study failed to be significantly associated with upgrading and thus the study was excluded (Supplementary Figure 1). Other studies similarly failed to find a significant association between these SNPs and aggressive PCa [47]. Study design or sample size may play a factor in this as these SNPs have well documented functional effects on tumourigenesis in PCa [48–50].

### Functional involvement of germline SNPs in disease progression

Post-GWAS functional studies aim to show that SNPs affect particular genes that may have a direct role in disease progression. Among the identified SNPs, 24 were located in intergenic regions, while 29 resided in either long noncoding RNAs or protein coding genes. Rs7652331 and rs1058205 were located in exons of genes (Table 1). Functional studies conducted on 21 (Table 1) of these 53 SNPs (Supplementary Table 1) were used to provide further evidence of their role in PCa progression. These 21 SNPs may drive aggressive PCa by acting through the oncogene *MYC*, the androgen receptor (AR) pathway, or through modulating known PCa biomarkers such as *MSMB* or *KLK3*, and genes involved in invasion, proliferation, suppression or metastasis.

**Table 1:** Functional information of 21 risk alleles identified to be associated with aggressive PCa

SNP	Location	Location of SNP – gene/Intergenic	Nearest coding gene/gene effected	Potential SNP function
rs13385191	2p24.1	intron *LDAH*	*LDAH*	down-regulates expression of *LDAH –* which is down-regulated in PCa and further reduced in metastatic prostate tumors versus primary prostate tumors [61]
rs2660753	3p12.1	Intergenic	*VGLL3/CHMP2B/POU1F1*	SNP associated with the expression of *VGLL3/CHMP2B/POU1F1* [58]
rs17021918	4q22	intron *PDLIM5*	*BMPR1B*	eQTL shows associated with (or fine mapped significantly with expression of) BMPR1B which is AR regulated [53]
rs7679673	4q24	Intergenic	*TET2*	significantly reduced *TET2* expression, a predictor of disease progression and poor overall survival as binds to the androgen receptor [42]
rs2939244	5q14.3	Intergenic	*ARRDC3/LUCAT1*	variation in androgen receptor-binding site gene *ARRDC3* affects prostate cancer specific mortality [56]
rs9364554	6q25.3	intron *SLC22A3*	*SLC22A3*	functional studies associate this risk allele with decreased *SLC22A3* transcript abundance [59]
rs10486567	7p15.2	intron *JAZF1*	*JAZF1*	affects both NKX3-1 and FOXA-AR motifs in *JAZF1*. SNP results in a 39% increase in basal activity and a 28% fold-increase in androgen stimulated enhancer activity [59]
rs6465657	7q21.3	intron *LMTK2*	*LMTK2*	effects *LMTK2* expression between benign (n = 39) and malignant tissues (n = 21) (P = 0.002) [60]
rs6983267	8q24.21	intron LOC727677/CASC8	*POU5F1B/MYC*	long-range interaction with *MYC* in colorectal cancer [48]
rs1447295	8q24.21	intron CASC8/LOC727677	*POU5F1B/MYC*	interferes with both *MYC* and *POU5F1B*'s activities through cell proliferation studies in LNCaP and C4-2B PCa cell lines [50]
rs4242382	8q24.21	intergenic	*POU5F1B/MYC*	implicated in the expression of *MYC* and *POU5F1B* [50]
rs1571801	9q33.2	intron *DAP2IP*	*DAB2IP*	^*^decrease in *DAB2IP* expression induces metastatic prostate cancer in a tumour mouse model [67] and chemoresistance in human prostate cancer cell lines [68]
rs10993994	10q11.23	intron PARG/ TIMM23B	*MSMB*	associated with *MSMB* [57]
rs10896449	11q13.3	intergenic	*MYEOV/DUSP6*	interacts with *DUSP6* [62], which promotes invasion and proliferation [63]
rs11568818	11q22.2	intergenic	*MMP-7*	associated with increased *MMP7* expression [65], which mediates IL-17's function in promoting prostate carcinogenesis in mice [66]
rs9508016	13q12.2	intron *FLT1*	*FLT1*	^*^increased *FLT1* expression in prostate cancer [69]
rs11649743	17q12	intron *HNF1B*	*HNF1B*	potentially increases *HNF1B* gene expression [64]
rs62113212	19q13.33	intron *KLK3*	*KLK3/KLK2*	^*^gene encodes PSA
rs266870	19q13.33	intron LOC105372441	*KLK3*	^*^gene encodes PSA
rs1058205	19q13.33	exon *KLK3*	*KLK3*	^*^gene encodes PSA
rs2735839	19q13.33	intergenic	*KLK3*	modulates PSA levels [39]

List of risk alleles associated with aggressive PCa, their respective chromosome, cytoband, and the location of each within either a gene or in an intergenic region as well as the gene they affect. Previously reported functional studies have been included to support these risk alleles in the role they play in aggressive PCa.

^*^not shown to directly affect gene expression.

### MYC pathway

Of the three SNPs reported in three or more studies in this review (rs1447295, rs10993994, and rs2735839), rs1447295 was found to be significantly associated with unfavourable outcomes in men with Gleason 6 (3+3), PSA recurrence [44] and aggressive PCa [45, 51, 41] (Table 1). This SNP is located within noncoding RNA LOC727677 and next to *POU5F1B*, which in turn has the oncogene *MYC* as its neighbouring gene [48]. Cai *et al*, 2016 [50] showed that rs1447295, as well as other PCa risk alleles on 8q24, interfere with both *MYC* and *POU5F1B’s* activities, through cell proliferation studies in LNCaP and C4-2B PCa cell lines. *MYC* is an established oncogene [33, 52], while *POU5F1B* has been shown to play an important role in PCa progression [50]. *POU5F1B* is expressed in normal prostate tissue and is overexpressed in prostatic carcinoma, compared to normal prostatic tissue surrounding the carcinoma [49]. Furthermore, PCa cell lines that ectopically overexpress *POU5F1B* form fewer cell-cell junctions and exhibit significantly increased invasiveness *in vitro* [49]. Given the evidence of rs1447295 functionally altering expression of *MYC* and *POU5F1B* [50], its influence on prostate tumor progression and metastasis can be asserted. Adding to the involvement of risk alleles on *MYC*, our analysis also highlighted rs6983267, which has been shown in colorectal cancer to interact with *MYC* [48] and rs4242382, affecting the expression of *MYC* and *POU5F1B* [50].

### Androgen receptor pathway

There is evidence supporting a role for 4 SNPs in modulating the genes of the AR pathway. The first, rs17021918, shows an association with *BMPR1B* via eQTL, a gene regulated by *AR* [53]. The second risk allele implicated in this pathway is rs10486567, which is located in an intron of *JAZF1* found to affect both *NKX3-1* and *FOXA-AR* motifs in the *JAZF1* gene. This risk allele results in a 39% increase in basal activity and a 28% fold-increase in androgen stimulated enhancer activity [54] (Table 1). The third risk allele, rs7679673, was found to significantly reduce *TET2* expression [55], which normally binds to the androgen receptor. This results in the capability of predicting disease progression and poor overall survival. Lastly, rs2939244 results in the variation of the androgen receptor-binding site gene *ARRDC3* which affects prostate cancer specific mortality [56].

### PCa biomarkers

Risk alleles that interact with other genes known to be PCa biomarkers were also identified. Rs10993994 on 10q11 is proximal to the promoter region of *MSMB*, a urinary biomarker that outperforms urinary PSA [57] at differentiating men with prostate cancer at all Gleason grades. An association between rs10993994's and levels of MSMB in prostate tissue was demonstrated, with levels of MSMB lowest in men homozygous for the high-risk allele (TT) and highest in men homozygous for CC [57].

Rs2735839 is located 600bp downstream of the PSA encoding *KLK3* gene, and was found to modulate PSA levels [39]. The clinical relevance of this risk allele is supported by the observation that rs2735839 is associated with biopsy-proven aggressive PCa (Gleason ≥8) and could stratify Gleason 7 patients [39]. Three additional SNPs were identified in either the intron (rs62113212 and rs266870) or exon (rs2735839) of *KLK3*.

### Tumourigenic genes

Nine risk alleles were found to modulate other tumourigenic genes. Genetic and functional analysis conducted by Grisanzio *et al*, [58] elucidated the functional relevance of both rs2660753 and rs9364554. Rs2660753 was found to be associated with the expression of *VGLL3/CHMP2B/POU1F1*. While rs9364554 was found to be associated with a decreased *SLC22A3* transcript abundance [58], and found to be down regulated in familial esophageal squamous cell carcinoma [59]. Other risk alleles have been shown to affect gene expression, such as rs6465657 on *LMTK2* expression [60], a gene implicated in the development of prostate cancer [60]. Rs13385191, which is suggested to be a cis-acting expressed quantitative locus (eQTL) that down-regulates the expression of *LDAH* [61], a gene that is frequently down-regulated in PCa and is even further reduced in metastatic prostate tumors as compared to primary prostate tumors [61]. Rs10896449 likely interacts with *DUSP6* [62], a gene that when knocked down promotes the invasion and proliferation of LNCap human prostate adenocarcinoma cells [63]. Rs11649743 potentially increases gene expression of *HNF1B* [64] and rs11568818, which is associated with increased *MMP7* expression [65], a gene which mediates IL-17's function in promoting prostate carcinogenesis in mice [66]. Two SNPs, reported as being significantly associated with aggressive PCa, reside in introns of genes, with their functional effect not yet determined, such as Rs1571801 located in the intron of *DAP2IP*. This gene is instrumental in tumourigenesis as it's loss induces metastatic prostate cancer in an orthotopic mouse tumour model [67] and chemoresistance in human prostate cancer cell lines [68]. Rs9508016 is located within the intron of *FLT1*, a gene which has been shown to promote angiogenesis and metastasis [69].

### Somatic copy number aberrations in PCa

Next, we hypothesised that the functional importance of these risk allele hotspots might be further elucidated if their somatic copy number aberrations (SCNAs) were altered in the genome. We evaluated the copy number data collected from different populations of low-grade and more aggressive PCa to see if this correlated with the SNPs we had identified. Seventeen of 53 SNPs were found in regions with SCNAs, such as a copy number gain on 8q24 encompassing rs1447295, rs10090154, rs6983267, rs4242382, rs16901966, rs6983561, rs6470517, and rs6999921 and gain on 11q13 where rs10896449 and rs11228565 reside (Table 2).

**Table 2:** SNPs found in recurrent somatic copy number aberrations or within 5 gene signatures predicting PCa aggressiveness

Location	SNP	Somatic copy number aberration (SCNA)	Gene from gene signature
**3p**	rs2660753, rs7652331, rs1545985,	gain 3p	-
**5q14.3**	rs35148638, rs2939244	loss 5q	-
**6q25.3**	rs9364554	loss 6q	-
**8p21.2**	rs1512268	loss 8p22	-
**8q24.21**	rs6983267, rs1447295, rs4242382, rs10090154	gain 8q24	-
**11q13.3**	rs10896449, rs11228565	gain 11q13	TMEM132A [83]
**13q12.2**	rs9508016	loss 13q	ZIC2, ZIC5 [87]
**17q**	rs11649743, rs6504145, rs1859962	loss 17q	-
**19q13**	rs11672691, rs62113212, rs266870, rs1058205, rs2735839, rs103294	-	KEAP1 [86], UPK1A [85], APOC1 [83]
**20q13.33**	rs2427345	-	MYBL2, UBE2C [84]

Table containing a subset of genes from 5 gene signatures predicting PCa aggressiveness, Wu et al [84], Ross et al [86], Bibikova et al [88], and Sahabi et al [85]. The respective chromosome arm and when applicable, cytoband for each gene along with recurrent SCNAs associated with PCa [21] within the same locus. A subset of SNPs associated with aggressive PCa within the same locus are also shown.

### Recurrent SCNAs indicative of aggression and metastases

Since PCa is predominately driven by DNA copy number loss and recurrent gains [70, 71], we hypothesise that SCNAs in low-grade PCa could be indicative for eventual metastasis or progression. SCNAs that predict PSA recurrence after radical prostatectomy include: a loss of *PTEN* [72], a simultaneous loss of *PTEN, FAS* (10q23.31) and *PAPSS2* (10q23.2–10q23.31) [73], a gain on 11q13.1 [74], a loss within 6q, a gain within 7q, a loss of 13q [75], a loss of 16q with or without a loss of *PTEN* [76], a gain of *MYC* [77], and a concurrent loss of 8p22 and gain of 8q24 [70]. SCNAs in certain genomic locations increase in frequency with Gleason score. For example, a loss of 8p22 was detected in ~30% Gleason score 7 [78], 69% in high-grade clinically organ-confined PCa (Gleason >8) and increased to 100% in metastatic patients (Gleason score 10) [79–81].

### SCNAs indicative of aggressiveness present in low-grade PCa

As the majority of SCNA studies have been conducted on high-grade PCa we sought to identify whether or not these were present and associated with an aggressive phenotype in low-grade PCa. A pooled low-grade PCa study found such SCNAs to be present, but at lower frequencies (>20%) than their higher-grade counterparts. These included a loss within 6q (location of rs9364554), 8p, 10q23, 13q, and 18q and a gain within 8q [71]. Another low-grade SCNA study conducted by Fraser *et al*, [20] also observed these SCNAs at similar frequencies in ~25% of Gleason 6 PCa samples, suggesting that men with PCa displaying those aberrations may have an aggressive form of the disease, and that these events occur early on in the development of cancer providing further evidence of punctuated evolution.

Of the SCNAs associated with aggressive PCa in high-grade PCa, evidence of a gain within 8q, loss of PTEN and 8p were found to also be associated with an aggressive phenotype in low-grade PCa [78]. In this study, differences in *PTEN* loss, a gain of chromosome 8q (encompassing *MYC*) and/or 8p loss (encompassing *LPL on* 8p22) were found in patients with only a Gleason pattern 3 versus those who had both Gleason pattern 3 and 4. Similar SCNAs, such as a loss of 8p, were associated with high-grade PCa and biochemical recurrence after radical prostatectomy [82]. In addition, Trock *et al*, (2016) [78] discovered that a gain on *MYC*/8q was a feature of undersampling a higher Gleason score tumour and was shown to be predictive of systematic disease progression, earlier PCa-specific death and PSA recurrence after radical prostatectomy in other studies [82]. Moreover, this *MYC*/8q SCNA encompasses eight SNPs reported in this review (Supplementary Table 1). Furthermore, a loss of *PTEN* was not only significantly associated with undersampling (p=0.03, conditional logistic regression) by Trock *et al*, (2016) but was also predictive of PSA recurrence after radical prostatectomy [72].

### Gene expression reflective of copy number aberrations

We next assessed whether genes from published gene signatures are encoded in genomic regions in which a) the identified germline SNPs reside; or b) SCNAs predictive of aggressive PCa were located. We collected data from five recently published PCa gene expression studies investigating progression, and recurrence along with the location of each gene (Table 2). These gene signatures were developed either from recurrent cohorts [83–85], by comparing high to low-grade PCas [86], or to predict upgrading upon repeat biopsies of Gleason 6 cohorts [87]. From a total of 95 genes across all five signatures, only 2 genes *ABCC11* and *HOXC6* were found in common. *ABCC11* expression was part of a gene signature predictive of recurrence [84] and between low to high grade PCas [86]. *HOXC6*, was found in both a signature indicative of recurrence [85] and a signature delineating low and high grade PCas [86]. Despite the limited number of overlapping genes between the signatures, we observed that the genomic position of risk alleles frequently coincides with genes from these signatures (Table 2). Ten of the 53 SNPs were in regions where the expression of genes was used in gene signatures of aggression. For example, *CCNE2* located on 8q22 is upstream of three identified SNPs, and *UPK1A* and *APOC1* reside on 19q13 where rs11672691, rs62113212, rs266870, rs1058205, rs2735839, rs103294, can be found (Table 2). In addition, several genes were found in regions of SCNAs that predict aggressive progression of PCa, such as *TMEM132A* on 11q12, which is next to a common SCNA on 11q13, and *ZIC2, ZIC5, F10* are located on 13q, a frequently lost SCNA region.

## DISCUSSION

This review collated GWAS studies that associated risk alleles with aggressive PCa, and included studies with patients who upgraded from Gleason 6 and patients with Gleason 6 and unfavourable histopathology. Twenty-nine potential germline GWAS ‘hotspots’ indicative of aggressive disease progression were identified containing 53 SNPs. These were then further assessed for their potential functional relevance. Of these, 17/53 were found in SCNAs associated with aggressive PCa and present to a lesser extent in low-grade PCa. Ten out of the 53 were found in gene expression signatures associated with PCa progression, further implicating the role these SNPs may have in aggressive tumourogenesis.

While SNPs are important for risk predictions in cancer, a singular SNP found to be associated to a disease through GWAS rarely has a sole effect on the disease. Most likely there is a complex mechanism underlying its effect. The use of functional studies conducted on 21 of the 53 risk alleles in combination with somatic genomic perturbations such as copy number status and gene expression levels provides further evidence that these regions play integral roles in the aggressive progression of PCa. Our work has implications in more than one population. Nine of the 53 identified SNPs identified were significantly associated with aggressive disease in more than one population.

We successfully identified SNPs within 29 loci. Rs1447295 was among 8 SNPs in the 8q24 hotspot and was found to be significantly associated with aggressive PCa in three studies [45, 44, 41], and adverse pathology in one low-grade GWAS study [37]. The effect of rs1447295 modulating *MYC* could be heightened by a gain or amplification at this locus, indicating the selective susceptibility of this hotspot to aggressive progression of the disease. Similar genomic hotspots of germline and somatic aberration alignments have been noted in breast cancer where tandem duplications were found to peak where two germline susceptibility loci were present on *MYC* [88]. Such regions are more prone to double stranded breaksand rely on faulty repair mechanisms that generate large tandem duplications [88]. We postulate that these hotspots and SCNAs, which are present in ~25% of patients with low-grade PCas [20], could be used to select patients whose disease might progress towards a more aggressive phenotype, deeming them worthy for further investigation in a clinical setting.

Limitations of this review include the GWAS and SCNA studies lack of consistent methodological reporting between studies examining low-grade PCa. Patients with a lower Gleason scoring PCa (<either Gleason score 6 or Gleason score 7) were compared to patients with more aggressive PCas in six case-case GWAS studies. However, only one study [42] in which the follow-up years of non-aggressive PCa remaining indolent was available. Another limitation stems from uncertainty around the significance of gene expression signatures. Gene expression fluctuates rapidly, rendering these gene expression patterns plastic throughout the life of any cancer. Therefore, combining low-grade and aggressive PCa expression signatures has its weaknesses. Acknowledging the limitations inherent in the available data, cross-referencing the different domains of risk alleles, SCNA and gene expression might help to overcome such uncertainties and identify key features for disease progression in PCa.

More than one in five men eligible for AS will present evidence of more aggressive disease upon prostatectomy [89]. Conversely, some men who do not meet conventional AS criteria may in fact harbour indolent cancers that will not reduce their length or quality of life. Furthermore, the current AS monitoring paradigm relies heavily on invasive and costly re-biopsy, with its concomitant risks and side effects. As such, there is a powerful argument for further investigation into non-invasive tests. In recent years, multiparametric MRI (mpMRI) has emerged as a potential non-invasive tool to detect clinical significant disease in men with low or intermediate risk of PCa [90]. Multi-platform non-invasive testing that integrates imaging and genomics, such as the 53 SNPs identified in this study with mpMRI, could conceivably present an effective method of determining candidacy for AS, and subsequent risk.

## MATERIALS AND METHODS

We focused on three specific types of data to predict PCa progression: germline GWAS risk alleles, SCNAs, and tumour gene expression data. Germline risk alleles of interest were identified. Manually curated functional risk alleles, SCNA and gene expression studies were used to investigate the potential relevance of these loci.

For GWAS data, we searched Embase and Pubmed using the following MeSH search terms: PCa, prostatic neoplasm, GWAS, genome-wide, inherited, aggressive, progression, active surveillance, death, and outcome for the period 2009-2017. We included GWAS studies, which either validated SNPs previously associated with PCa or compiled information regarding recurrence or progression of the disease. For each study, we assessed risk markers in their role of developing aggressive PCas, which may result in metastasis, biochemical recurrence, and death. Of 698 articles assessed, 24 were carried forward (Supplementary Figure 1) using the PRISMA statement [91].

To corroborate GWAS hotspots, research on SCNAs and gene expression profiles were identified in PubMed, until January 2017, using the search terms listed above as well as copy number and gene expression. We specifically included those studies with somatic copy number data derived from microarrays or DNA sequencing analysis, gene expression profiles from expression microarrays and RNA sequencing, and clinical information, including recurrence or progression of disease.

## SUPPLEMENTARY MATERIALS FIGURE AND TABLE





## Abbreviations

AS: Active surveillance
PCa: Prostate cancer
PSA: Prostate specific antigen
GWAS: Genome-wide association study
SNP: Single nucleotide polymorphism
SCNA: Somatic copy number aberration
