# Supplementary material for: Systematic identification of functionally relevant risk alleles to stratify aggressive versus indolent prostate cancer
Source: Oncotarget. 2018 Feb 5;9(16):12812–24. doi: 10.18632/oncotarget.24400 (PMC5849176; doi:10.18632/oncotarget.24400)
Supplement: Supplementary file 1 [file oncotarget-09-12812-s001.pdf]

Systematic identification of functionally relevant risk alleles to stratify aggressive versus indolent prostate cancer

SUPPLEMENTARY MATERIALS

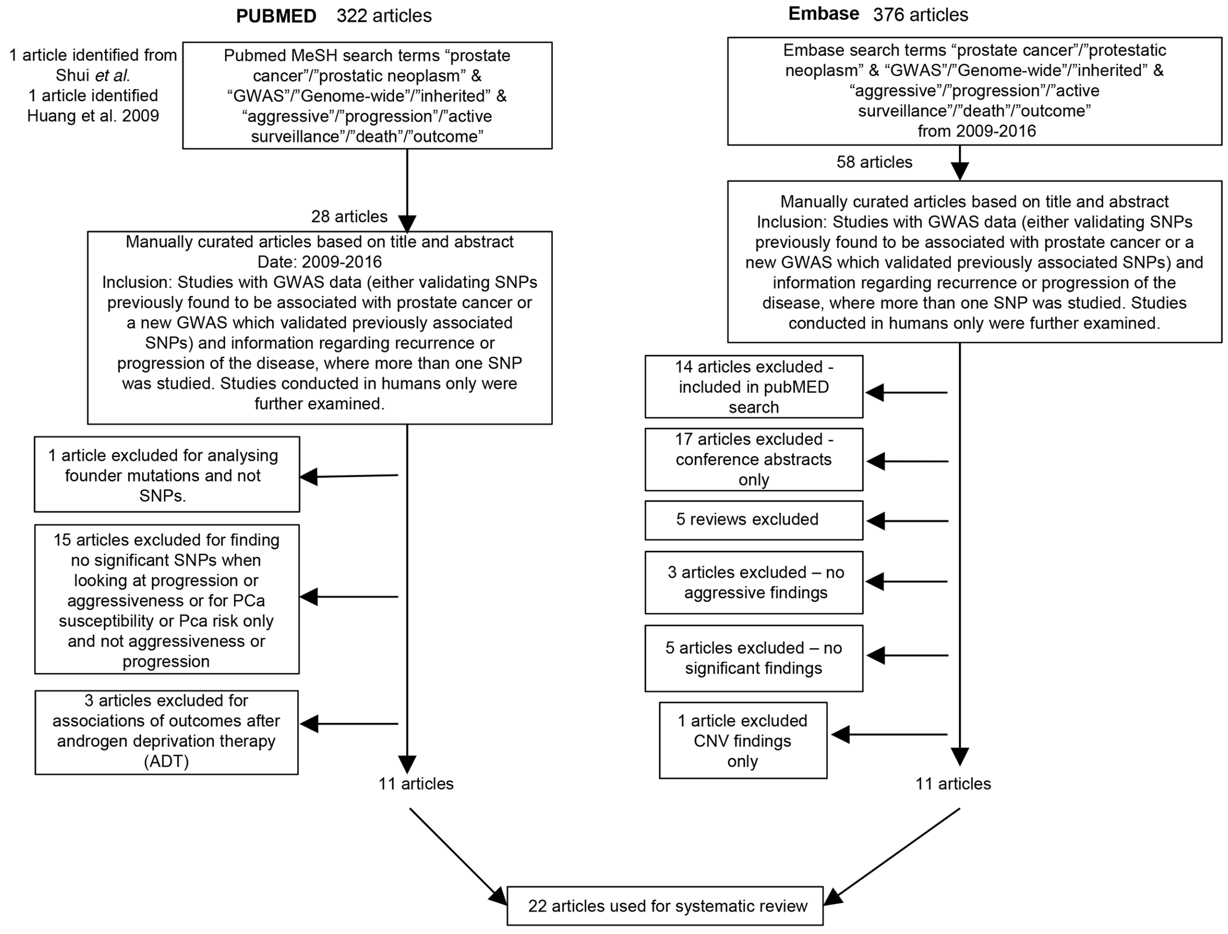

Supplementary Figure 1: Flow chart of evidence acquisition and synthesis GWAS articles were excluded based on whether or not any significant SNPs were found to be associated with aggressive PCa, or progressive PCa, and included SNPs not founder mutations. Articles that passed these criteria were then pooled from Embase and Pubmed and used for the systematic review according to PRISMA guidelines.

Supplementary Table 1: SNPs significantly associated with aggressive PCa

See Supplementary File 1

REFERENCES

1. Shui IM, Lindström S, Kibel AS, Berndt SI, Campa D, Gerke T, Penney KL, Albanes D, Berg C, Bueno-de-Mesquita HB, Chanock S, Crawford ED, Diver WR, et al. Prostate cancer (PCa) risk variants and risk of fatal PCa in the National Cancer Institute Breast and Prostate Cancer Cohort Consortium. Eur Urol. 2014; 65:1069–75. https://doi.org/10.1016/j.eururo.2013.12.058.
2. Cheng I, Plummer SJ, Neslund-Dudas C, Klein EA, Casey G, Rybicki BA, Witte JS. Prostate cancer susceptibility variants confer increased risk of disease progression. Cancer Epidemiol Biomarkers Prev. 2010; 19:2124–32. https://doi.org/10.1158/1055-9965.EPI-10-0268.
3. Bensen JT, Xu Z, Smith GJ, Mohler JL, Fontham ET, Taylor JA. Genetic polymorphism and prostate cancer aggressiveness: a case-only study of 1,536 GWAS and candidate SNPs in African-Americans and European-Americans. Prostate. 2013; 73:11–22. https://doi.org/10.1002/pros.22532.
4. Berndt SI, Wang Z, Yeager M, Alavanja MC, Albanes D, Amundadottir L, Andriole G, Beane Freeman L, Campa D, Cancel-Tassin G, Canzian F, Cornu JN, Cussenot O, et al, and African Ancestry Prostate Cancer GWAS Consortium. Two susceptibility loci identified for prostate cancer aggressiveness. Nat Commun. 2015; 6:6889. https://doi.org/10.1038/ncomms7889.
5. Agalliu I, Wang Z, Wang T, Dunn A, Parikh H, Myers T, Burk RD, Amundadottir L. Characterization of SNPs associated with prostate cancer in men of Ashkenazic descent from the set of GWAS identified SNPs: impact of cancer family history and cumulative SNP risk prediction. PLoS One. 2013; 8:e60083. https://doi.org/10.1371/journal.pone.0060083.
6. Huang CN, Huang SP, Pao JB, Chang TY, Lan YH, Lu TL, Lee HZ, Juang SH, Wu PP, Pu YS, Hsieh CJ, Bao BY. Genetic polymorphisms in androgen receptor-binding sites predict survival in prostate cancer patients receiving androgen-deprivation therapy. Ann Oncol. 2012; 23:707–13. https://doi.org/10.1093/annonc/mdr264.
7. He Y, Gu J, Strom S, Logothetis CJ, Kim J, Wu X. The prostate cancer susceptibility variant rs2735839 near KLK3 gene is associated with aggressive prostate cancer and can stratify gleason score 7 patients. Clin Cancer Res. 2014; 20:5133–39. https://doi.org/10.1158/1078-0432.CCR-14-0661.
8. Ahn J, Kibel AS, Park JY, Rebbeck TR, Rennert H, Stanford JL, Ostrander EA, Chanock S, Wang MH, Mittal RD, Isaacs WB, Platz EA, Hayes RB. Prostate cancer predisposition loci and risk of metastatic disease and prostate cancer recurrence. Clin Cancer Res. 2011; 17:1075–81. https://doi.org/10.1158/1078-0432.CCR-10-0881.
9. McGuire BB, Helfand BT, Kundu S, Hu Q, Banks JA, Cooper P, Catalona WJ. Association of prostate cancer risk alleles with unfavourable pathological characteristics in potential candidates for active surveillance. BJU Int. 2012; 110:338–43. https://doi.org/10.1111/j.1464-410X.2011.10750.x.
10. Terada N, Tsuchiya N, Ma Z, Shimizu Y, Kobayashi T, Nakamura E, Kamoto T, Habuchi T, Ogawa O. Association of genetic polymorphisms at 8q24 with the risk of prostate cancer in a Japanese population. Prostate. 2008; 68:1689–95. https://doi.org/10.1002/pros.20831.
11. Huang SP, Huang LC, Ting WC, Chen LM, Chang TY, Lu TL, Lan YH, Liu CC, Yang WH, Lee HZ, Hsieh CJ, Bao BY. Prognostic significance of prostate cancer susceptibility variants on prostate-specific antigen recurrence after radical prostatectomy. Cancer Epidemiol Biomarkers Prev. 2009; 18:3068–74. https://doi.org/10.1158/1055-9965.EPI-09-0665.
12. Teerlink CC, Thibodeau SN, McDonnell SK, Schaid DJ, Rinckleb A, Maier C, Vogel W, Cancel-Tassin G, Egrot C, Cussenot O, Foulkes WD, Giles GG, Hopper JL, et al, and International Consortium for Prostate Cancer Genetics. Association analysis of 9,560 prostate cancer cases from the International Consortium of Prostate Cancer Genetics confirms the role of reported prostate cancer associated SNPs for familial disease. Hum Genet. 2014; 133:347–56. https://doi.org/10.1007/s00439-013-1384-2.
13. Liu M, Wang J, Xu Y, Wei D, Shi X, Yang Z. Risk loci on chromosome 8q24 are associated with prostate cancer in northern Chinese men. J Urol. 2012; 187:315–21. https://doi.org/10.1016/j.juro.2011.09.011.
14. Chen M, Huang YC, Yang S, Hsu JM, Chang YH, Huang WJ, Chen YM. Common variants at 8q24 are associated with prostate cancer risk in Taiwanese men. Prostate. 2010; 70:502–07. https://doi.org/10.1002/pros.21084.
15. Pal P, Xi H, Guha S, Sun G, Helfand BT, Meeks JJ, Suarez BK, Catalona WJ, Deka R. Common variants in 8q24 are associated with risk for prostate cancer and tumor aggressiveness in men of European ancestry. Prostate. 2009; 69:1548–56. https://doi.org/10.1002/pros.20999.
16. Nam RK, Zhang W, Siminovitch K, Shlien A, Kattan MW, Klotz LH, Trachtenberg J, Lu Y, Zhang J, Yu C, Toi A, Loblaw DA, Venkateswaran V, et al. New variants at 10q26

and 15q21 are associated with aggressive prostate cancer in a genome-wide association study from a prostate biopsy screening cohort. Cancer Biol Ther. 2011; 12:997–1004. https://doi.org/10.4161/cbt.12.11.18366.

17. Kearns JT, Lapin B, Wang E, Roehl KA, Cooper P, Catalona WJ, Helfand BT. Associations Between iCOGS Single Nucleotide Polymorphisms and Upgrading in Both Surgical and Active Surveillance Cohorts of Men with Prostate Cancer. Eur Urol. 2016; 69:223–28. https://doi.org/10.1016/j.eururo.2015.09.004.
18. FitzGerald LM, Kwon EM, Conomos MP, Kolb S, Holt SK, Levine D, Feng Z, Ostrander EA, Stanford JL. Genome-wide association study identifies a genetic variant associated with risk for more aggressive prostate cancer. Cancer Epidemiol Biomarkers Prev. 2011; 20:1196–203. https://doi.org/10.1158/1055-9965.EPI-10-1299.
19. Xu J, Zheng SL, Isaacs SD, Wiley KE, Wiklund F, Sun J, Kader AK, Li G, Purcell LD, Kim ST, Hsu FC, Stattin P, Hugosson J, et al. Inherited genetic variant predisposes to aggressive but not indolent prostate cancer. Proc Natl Acad Sci USA. 2010; 107:2136–40. https://doi.org/10.1073/pnas.0914061107.
20. Amin Al Olama A, Kote-Jarai Z, Schumacher FR, Wiklund F, Berndt SI, Benlloch S, Giles GG, Severi G, Neal DE, Hamdy FC, Donovan JL, Hunter DJ, Henderson BE, et al, and UK Genetic Prostate Cancer Study Collaborators/British Association of Urological Surgeons' Section of Oncology, and UK ProtecT Study Collaborators, and Australian Prostate Cancer Bioresource, and PRACTICAL Consortium. A meta-analysis of genome-wide association studies to identify prostate cancer susceptibility loci associated with aggressive and non-aggressive disease. Hum Mol Genet. 2013; 22:408–15. https://doi.org/10.1093/hmg/dds425.
21. Helfand BT, Roehl KA, Cooper PR, McGuire BB, Fitzgerald LM, Cancel-Tassin G, Cornu JN, Bauer S, Van Blarigan EL, Chen X, Duggan D, Ostrander EA, Gwo-Shu M, et al. Associations of prostate cancer risk variants with disease aggressiveness: results of the NCI-SPORE Genetics Working Group analysis of 18,343 cases. Hum Genet. 2015; 134:439–50. https://doi.org/10.1007/s00439-015-1534-9.
22. Sun J, Zheng SL, Wiklund F, Isaacs SD, Li G, Wiley KE, Kim ST, Zhu Y, Zhang Z, Hsu FC, Turner AR, Stattin P, Liu W, et al. Sequence variants at 22q13 are associated with prostate cancer risk. Cancer Res. 2009; 69:10–15. https://doi.org/10.1158/0008-5472.CAN-08-3464.
