# Supplementary material for: Systematic identification of functionally relevant risk alleles to stratify aggressive versus indolent prostate cancer
Source: Oncotarget. 2018 Feb 5;9(16):12812–24. doi: 10.18632/oncotarget.24400 (PMC5849176; doi:10.18632/oncotarget.24400)
Supplement: Supplementary file 2 [file oncotarget-09-12812-s002.docx]

**Supplementary Table 1:** **SNPs significantly associated with aggressive PCa**

| SNP | OR/HR CI and p-value | location | gene/intergenic | Ancestry | Author |
| --- | --- | --- | --- | --- | --- |
| rs13385191 | 0.88 (0.78-1.00) (p=0.05) | 2p24.1 | intron LDAH | European Ancestry | [1] |
| rs12621278 | 2.43 (1.51-3.92) (p=0.00026) | 2q31.1 | intron ITGA6 | Mixed Population – White, African American | [2] |
| rs2660753 | 1.5(1.1-1.9) (p=0.007) (EA) | 3p12.1 | intergenic | African American and European American | [3] |
| rs7652331 | 1.39 (1.02-1.89) (p=0.036) | 3p21.31 | exon FYCO1 | Mixed Population – White, African American | [2] |
| rs1545985 | 1.38 (1.03-1.86) (p=0.034) | 3p21.31 | intron FYCO1 | Mixed Population – White, African American | [2] |
| rs78943174 | *(p=4.18x10-8) (combined Ancestrys) | 3q26.31 | intron NAALADL2 | European Ancestry | [4] |
| rs629242 | 1.63 (1.01-2.64) (p=0.047) | 4q12 | intron KIAA1211 | Mixed Population – White, African American | [2] |
| rs17021918 | 0.89 (0.81-0.97) (p=0.01) | 4q22 | intron PDLIM5 | European Ancestry | [1] |
| rs7679673 | 0.81 (0.69-0.94) (p=0.002) | 4q24 | intergenic | Ashkenazi | [5] |
| rs35148638 | *(p=6.49x10-8) (combined Ancestrys) | 5q14.3 | intron RASA1 | European Ancestry | [4] |
| rs2939244 | 2.07 (1.29-3.31) (p=0.002) | 5q14.3 | intergenic | Taiwan | [6] |
| **rs9364554** | 1.37 1.13-1.65 (p=0.001) | 6q25.3 | intron SLC22A3 | Ashkenazi | [5] |
|  | 1.27 (1.01-1.61) (p=0.041) |  |  | Mixed Population – White, African American | [2] |
| **rs10486567** | 1.54 (1.10-2.16) p=0.012 | 7p15.2 | intron JAZF1 | American | [7] |
|  | 0.85 (0.76-0.94) (p=0.001) | 7p15.3 |  | European Ancestry | [1] |
| rs12155172 | 1.83 (1.05-3.19) (0.034) | 7p15.3 | intron LINC01162 | Mixed Population – White, African American | [2] |
| rs6465657 | 0.90 (0.82-0.98) (p=0.02) | 7q21.3 | intron LMTK2 | European Ancestry | [1] |
| rs1512268 | 1.46 (1.02-2.08) (p=0.037) | 8p21.2 | intergenic | Mixed Population – White, African American | [2] |
| **rs6983267** | 1.36 (1.16-1.58) (p<0.0001) | 8q24.21 | intron LOC727677/CASC8 | Ashkenazi | [5] |
|  | 0.67 (0.50-0.89) (p=0.006) |  |  | Asian Indian and African Americans | [8] |
| **rs1447295** | * (p=0.004) | 8q24.21 | intron CASC8/LOC727677 | European Ancestry | [9] |
|  | 1.43 (1.08-1.90) (p=0.013) |  |  | Japanese Ancestry | [10] |
|  | 1.59 (1.08-2.35) (p=0.019) |  |  | Taiwan | [11] |
|  | 1.46 (1.27-1.69) (p<1x10-7) |  |  | Mixed Population - African, European, Australian, Canadian, US | [12] |
| **rs4242382** | 1.40 (1.13-1.75) (p=0.003) | 8q24.21 | intergenic | Asian Indian and African Americans | [8] |
|  | 1.39 (1.21-1.61) (p=4x10-6) |  |  | Mixed Population - African, European, Australian, Canadian, US | [12] |
| rs10090154 | 1.47 (1.27-1.7) (p<1x10-7) | 8q24.21 | intergenic | Mixed Population - African, European, Australian, Canadian, US | [12] |
| rs16901966 | 1.54 (1.08-2.10) (p=0.018) | 8q24.21 | intergenic | Chinese Ancestry | [13] |
| rs6983561 | 1.47 (1.12-1.89) (p=0.004) | 8q24.21 | intergenic | Taiwan | [14] |
| rs6470517 | 1.58 (1.28-1.96) (p=7.6×10−4) | 8q24.21 | intron CASC8/LOC727677 | European Ancestry | [15] |
| rs6999921 | 0.7 (0.5-0.9) (p =0.005) (AA)  1.3 (0.9-1.8) (p =0.05) (EA) | 8q24.21 | intron CASC8/LOC727677 | African American and European American | [3] |
| rs1571801 | * (p=0.03) | 9q33.2 | intron DAP2IP | European Ancestry | [9] |
| **rs10993994** | 1.26 (1.08-1.47) (p=0.002) | 10q11.23 | intron PARG/TIMM23B | Ashkenazi | [5] |
|  | 1.20 (1.08-1.33) (p=9.07x10-4) |  |  | Mixed Population - African, European, Australian, Canadian, US | [12] |
|  | 1.66 (1.19-2.31) p=0.003 |  |  | Taiwan | [11] |
|  | 0.90 (0.83-0.98) (p=0.02) |  |  | European Ancestry | [1] |
|  | 1.24 (1.05-1.48) (p=0.012) |  |  | Asian Indian and African Americans | [8] |
| rs7920517 | 1.59 (1.14-2.21) p=0.006 | 10q11.23 | intron PARG/TIMM23B | Taiwan | [11] |
| rs10749408 | *(p=6.9x10-6) | 10q26.12 | intergenic | North American Ancestry | [16] |
| rs11199874 | *(p=2.6x10-10) | 10q26.12 | intergenic | North American Ancestry | [16] |
| rs10788165 | * (p=1.2x10-7) | 10q26.12 | intergenic | North American Ancestry | [16] |
| rs7127900 | 0.86 (0.77-0.97) (p=0.01) | 11p15.5 | intergenic | European Ancestry | [1] |
| **rs10896449** | 1.28 (1.15-1.43) (p=7.2x10-6) | 11q13.3 | intergenic | Mixed Population - African, European, Australian, Canadian, US | [12] |
|  | 1.28 (1.04-1.56) (p=0.018) |  |  | Mixed Population – White, African American | [2] |
| rs11228565 | *(p=0.02) | 11q13.3 | intergenic | European Ancestry | [9] |
| rs11568818 | 1.4 (1.2-1.7) (p=0.0008) | 11q22.2 | intergenic | European Ancestry | [17] |
| rs9508016 | 1.82 (1.17-2.83) (p=0.008) | 13q12.2 | intron FLT1 | Taiwan | [6] |
| rs6497287 | 1.46 (1.10-1.94) (p=0.004) | 15q13.1 | intron HERC2 | Caucasian Ancestry | [18] |
| rs4775302 | * (p=4.1x10-8) | 15q21.1 | intergenic | North American Ancestry | [16] |
| rs1994198 | *(p=5.8x10-7) | 15q21.1 | intergenic | North American Ancestry | [16] |
| rs4054823 | 1.26 (1.16-1.36) (p=2.1x10-8) (combined Ancestry) | 17p12 | intergenic | European Ancestry | [19] |
| rs11649743 | 1.34 (1.17, 1.54) (p=4.08x10-5) | 17q12 | intron HNF1B | Mixed Population - African, European, Australian, Canadian, US | [12] |
| rs6504145 | 0.55 (0.32–0.96) (p=0.035) | 17q21.32 | intron SKAP1 | Taiwan | [6] |
| rs1859962 | 1.24 (1.12, 1.38) (p=5.48x10-5) | 17q24.3 | intron BC039327/CASC17 | Mixed Population - African, European, Australian, Canadian, US | [12] |
| **rs11672691** | 1.12 (1.03-1.21) (p=1.4x10-8) | 19q13.2 | intron LOC100505495/PCAT19 | European Ancestry | [20] |
|  | 1.18 (1.05–1.34) (p=0.007) |  |  | European Ancestry | [1] |
| rs62113212 | *(p=5.85x10-9) (combined Ancestrys) | 19q13.33 | intron KLK3 | European Ancestry | [4] |
| rs266870 | 0.8(0.7–1.0) (p=0.049) (AA)  1.2(1.0–1.5) (p=0.015) ) (EA) | 19q13.33 | intron LOC105372441 | African American and European American | [3] |
| rs1058205 | 0.8(0.6–0.9) (p=0.004) (AA) | 19q13.33 | exon KLK3 | African American and European American | [3] |
| **rs2735839** | 1.81 (1.23-2.65) (p=0.002) | 19q13.33 | intergenic | American | [7] |
|  | 0.77 (0.68–0.86) (p=1.862×10−5) (E)  0.69 (0.54–0.87) (p=4.667×10−4) (AA) |  |  | Mixed Population – African American and European | [21] |
|  | 0.82 (0.73–0.93) (p=0.002) |  |  | European Ancestry | [1] |
| rs103294 | 0.65 (0.45-0.95) (p=0.024) | 19q13.42 | intron LILRA6 | American | [7] |
| rs2427345 | 1.4 (1.1-1.7) (p=0.005) | 20q13.33 | intergenic | European Ancestry | [17] |
| rs11704416 | 0.95 (0.92-0.98) (p=3.7x10-7) | 22q13.1 | intergenic | European Ancestry | [20] |
| rs9623117 | 1.18 (1.11-1.26) (p=5.0x10-7) | 22q13.1 | intron TNRC6B | Mixed Population | [22] |
| rs5945619 | 1.28 (1.13-1.44) (p=4.8x10-5) | Xp11.22 | intergenic | Mixed Population - African, European, Australian, Canadian, US | [12] |
| rs5945572 | 1.45 (1.10-1.91) (p=0.008) | Xp11.22 | intergenic | Mixed Population – White, African American | [2] |

SNPs found to be significantly associated with aggressive PCa and their respective chromosomal cytoband, the hazard ratio (HR) or odds ratio (OR) along with confidence intervals (CI) and pvalues as stated in each article, the nearest protein coding gene, and the Ancestry the risk allele was found to be significant in. The “gene/intergenic” column indicates whether or not the risk allele was identified within the exon/intron of a gene or within an intergenic region. The most significant ORs, CIs, and associated pvalues stated as described in the respective articles. Ancestry specific ORs have been given stated for African American ancestry (AA), European American ancestry (EA) and European ancestry (E). In bold are those SNPs found to be significant in more than one article.

* No OR/HR or CI stated in article
